# Beyond reliability: assessing rater competence when using a behavioural marker system

**DOI:** 10.1186/s41077-024-00329-9

**Published:** 2024-12-31

**Authors:** Samantha Eve Smith, Scott McColgan-Smith, Fiona Stewart, Julie Mardon, Victoria Ruth Tallentire

**Affiliations:** 1 Centre for Medical Education, University of Dundee, Dundee, UK; 2 NHS Education for Scotland, Glasgow, UK; 3 Scottish Centre for Simulation and Clinical Human Factors, NHS Forth Valley, Larbert, UK; 4 Medical Education Directorate, NHS Lothian, Edinburgh, UK

## Abstract

**Background:**

Behavioural marker systems are used across several healthcare disciplines to assess behavioural (non-technical) skills, but rater training is variable, and inter-rater reliability is generally poor. Inter-rater reliability provides data about the tool, but not the competence of individual raters. This study aimed to test the inter-rater reliability of a new behavioural marker system (PhaBS — pharmacists’ behavioural skills) with clinically experienced faculty raters and near-peer raters. It also aimed to assess rater competence when using PhaBS after brief familiarisation, by assessing completeness, agreement with an expert rater, ability to rank performance, stringency or leniency, and avoidance of the halo effect.

**Methods:**

Clinically experienced faculty raters and near-peer raters attended a 30-min PhaBS familiarisation session. This was immediately followed by a marking session in which they rated a trainee pharmacist’s behavioural skills in three scripted immersive acute care simulated scenarios, demonstrating good, mediocre, and poor performances respectively. Inter-rater reliability in each group was calculated using the two-way random, absolute agreement single-measures intra-class correlation co-efficient (ICC). Differences in individual rater competence in each domain were compared using Pearson’s chi-squared test.

**Results:**

The ICC for experienced faculty raters was good at 0.60 (0.48–0.72) and for near-peer raters was poor at 0.38 (0.27–0.54). Of experienced faculty raters, 5/9 were competent in all domains versus 2/13 near-peer raters (difference not statistically significant). There was no statistically significant difference between the abilities of clinically experienced versus near-peer raters in agreement with an expert rater, ability to rank performance, stringency or leniency, or avoidance of the halo effect. The only statistically significant difference between groups was ability to compete the assessment (9/9 experienced faculty raters versus 6/13 near-peer raters, *p* = 0.0077).

**Conclusions:**

Experienced faculty have acceptable inter-rater reliability when using PhaBS, consistent with other behaviour marker systems; however, not all raters are competent. Competence measures for other assessments can be helpfully applied to behavioural marker systems. When using behavioural marker systems for assessment, educators must start using such rater competence frameworks. This is important to ensure fair and accurate assessments for learners, to provide educators with information about rater training programmes, and to provide individual raters with meaningful feedback.

**Supplementary Information:**

The online version contains supplementary material available at 10.1186/s41077-024-00329-9.

## Background

Behavioural marker systems (BMS) are used across several healthcare disciplines to assess behavioural (non-technical) skills, but rater training is very variable, and inter-rater reliability (Table 1) is generally poor [1]. BMS, such as ANTS (anaesthetists non-technical skills) [2], NOTSS (non-technical skills for surgeons) [3], and Medi-StuNTS (medical students non-technical skills) [4], have been incorporated into formal training curricula. However, if inter-rater reliability is poor, we are left with the problem of deciding when raters are ‘good enough’ and which raters to choose.

**Table 1:** Definitions [5]

Inter-rater reliability: “The extent to which independent evaluators produce similar ratings in judging the same abilities or characteristics in the same target person or object” [5]
Rater competence: Raters’ ability to use the tool to differentiate between different levels of performance across different skills, both between participants and between one individual’s skills in various domains

According to Messick’s validity framework [6], inter-rater reliability is part of the evidence often provided when examining the internal structure of an assessment tool [7]. Evidence of good reliability is essential for summative assessments but much less important for formative assessments [8]. Where assessments rely on human raters, the greatest threat to the reliability of the assessment is rater inconsistency [9]. It is therefore very important to study inter-rater reliability when introducing a new assessment tool into a curriculum, particularly if it is to be used as part of a high-stakes summative examination. Inter-rater reliability is also crucially important if an assessment tool is to be used as part of research.

However, while inter-rater reliability can provide some validity evidence for a tool, it does not provide any evidence with regard to the competence of individual raters (see definition, Table 1). Information about rater competence could be helpful for providing individualised and meaningful feedback to raters, as well as informing evaluation of rater training programmes.

Individual rater competence depends on several factors. Generally speaking, the extent to which a rater agrees with the goals and methods of the training assessment will influence their accuracy [10]. Yeates et al. [11] argue that variability between raters is due to three factors: differential salience (where raters place focus or emphasis on different factors); criterion uncertainty (where raters are unsure of the standards expected of a certain level of learner); and information integration (when raters use their own narrative language to assess a learner and then convert this to the given scale afterwards) [11]. Within BMS ratings specifically, raters may misclassify behaviours into the wrong elements, especially when using an unfamiliar BMS [12].

There are several methods of assessing rater competence. At the simplest level, we can assess whether raters are able to fully complete the assessment for each participant. It has also been proposed that rater competence assessments could include agreement with another rater [13], ability to rank participants in order of performance [14], and assessments of stringency versus leniency (whether a candidate is a ‘hawk’ or a ‘dove’) [15]. A further source of rater error is the so-called halo effect [16], in which a rater makes a global judgement about a participant and uses this to inform all ratings, instead of discriminating between different behaviours. This error may be particularly relevant for BMS, but has not yet been discussed in the simulation literature.

In the current study, we set out to assess the inter-rater reliability of a new BMS, called PhaBS (pharmacists’ behavioural skills) [17]. When considering inter-rater reliability, we noted that previously reported figures for other BMS were often low [1], even after 8 h of rater training [12]. Previous work suggested that clinically experienced faculty were more likely to have good inter-rater reliability than clinically inexperienced raters [1]. Possible reasons for this include experts paying more attention to contextual factors [18] and noticing cues that novices tend to ignore [19].

However, even with good inter-rater reliability, we wanted to understand how many of the raters could be considered competent to use the BMS. This information would facilitate recommendations about the use of PhaBS within training curricula and research, as well as providing frameworks by which researchers could investigate the competence of their BMS raters.

### Aims

#### Main aim

To test the inter-rater reliability of the PhaBS marker system with both clinically experienced faculty raters and near-peer raters.

#### Secondary aim

To assess the overall competence of raters to use the PhaBS marker system after brief familiarisation, by assessing the following characteristics:

1. Completeness of the data
2. Agreement with an expert rater
3. Ability to rank performances
4. Stringency or leniency
5. Ability to identify an appropriate behaviour range (avoiding the ‘halo effect’)

#### Relevance to the simulation community

This study may be of particular relevance to the simulation community, as simulation is an effective tool to aid learning of behavioural skills [20], and learning outcomes relating to behavioural skills are common within simulation. The Healthcare Simulation Standards of Best Practice encourage the use of measurable objectives and suggest building simulation scenarios to align with these objectives [21]. BMS may therefore provide a framework for scenario design, with different scenarios aligned to different behavioural skills identified within the BMS. BMS may also be useful within debriefing, either as an aid for self-debriefing [22] or to provide a common language for facilitators and learners to discuss their observations and experiences [23]. Finally, BMS may also be used as observation tools for learners who are not actively participating within scenarios, because evidence suggests that observer tools improve learning and satisfaction for such participants [24].

## Methods

### Ethical approval

Ethical approval was granted by the NHS Education for Scotland Research Ethics Service (NES/Res/30/22/Pharm).

### Study design

We created three videos of simulated scenarios in which a trainee pharmacist (pharmacist in their first year after qualification), working together with other staff, treated a sick patient while displaying differing levels of trainee pharmacist behavioural skills. We then recruited pharmacist raters to participate in a 30-min PhaBS familiarisation session, immediately followed by independently rating the trainee pharmacists’ behavioural skills in the videos using PhaBS.

### Context and setting

This study took place in Scotland, where pharmacist training involves a 4-year undergraduate master’s degree, followed by 1 year in which they are employed and supervised as a trainee pharmacist [25]. Within hospitals, pharmacists play a key role as part of a multidisciplinary team and are involved in the treatment of acutely unwell patients. Trainee pharmacists in Scotland undertake interprofessional immersive simulations to help them to learn the behavioural skills required to help them to perform these tasks. PhaBS is not yet incorporated as an assessment tool, but there are plans to trial it as a formative assessment tool within the new Post-Registration Foundation Simulation Programme scheduled to launch in Summer 2025. There are also plans to introduce PhaBS as a research tool, hence the focus on inter-rater reliability in this paper.

### The PhaBS tool

PhaBS shares the structure of all BMS, which include overarching categories, elements within those categories, and observable positive and negative behaviours. PhaBS categories and elements are shown in Table 2. A full version of the tool, and the content validity evidence, has been published previously [17].

**Table 2:** Categories (headers) and elements (under each category) within the PhaBS BMS

Situation awareness	Decision-making and prioritisation	Collaborative working	Self-awareness
Gathering information	Identifying options	Involving the patient	Role awareness
Recognising and understanding information	Prioritising	Information sharing	Speaking up
Anticipating, preparing and planning	Dealing with uncertainty	Leadership or followership	Escalating care
	Implementing or reviewing decisions		Coping with stress

If used summatively, each element of PhaBS is scored as either 1 (poor), 2 (marginal), 3 (acceptable), 4 (good), or 5 (excellent). ‘Poor’ behaviours are those which threaten patient safety, and ‘excellent’ behaviours are positive examples for others. This scoring system echoes that of other BMS [2–4].

### Choice of simulation as a research tool

Within simulation-based research, studies can assess the efficacy of simulation (simulation as the *object* of the research) or can use simulation as an investigative methodology (simulation as the *tool* for the research) [26]. This study used simulation as the tool for the research, rather than using real-life scenarios because it was possible to script the scenarios to demonstrate a variety of positive and negative behaviours and also possible for senior clinicians to observe poor behaviours without intervening. However, while simulation was the tool for the research, the results of this research may be of particular relevance to the simulation community, as explained above.

### Scenarios

We produced three videos of immersive simulated scenarios. Each scenario consisted of a pharmacist (acting at the level of a trainee pharmacist) working with an advanced nurse practitioner (acting at the level of a junior doctor) to treat an acutely unwell patient (SimMan or actor). The same pharmacist acted in each video, and the videos were scripted in order to demonstrate both positive and negative behaviours. One performance was overall good, with mostly positive behaviours, one was overall poor with mostly negative behaviours, and one was mediocre, with a mixture of positive and negative behaviours. However, even within the poor performance, some acceptable behaviours were still evident.

The patient observations were controlled remotely and available on the patient’s bedside monitor. The three scenarios included an elderly patient with urosepsis, a patient with severe acute asthma, and a patient with epilepsy who had suffered a stroke. Further details of the patient scenarios are given in the Additional file 1: Appendix. Scenarios lasted between 5 and 6 min.

### Rater recruitment

We recruited raters by email. Raters were identified by the principal lead for pharmacy simulation, and were all either clinically experienced members of pharmacy simulation faculty, or near-peer pharmacists (qualified within the last 3 years). We excluded faculty who had been involved in the development of PhaBS. Participation was voluntary, and raters provided informed written consent.

We based our sample size on the Medi-STuNTS inter-rater reliability study [1], which demonstrated that 11 clinically experienced raters were required to achieve good inter-rater reliability. We aimed for 12 raters in each group, to account for unexpected dropouts. We expected this to give us a narrow width for the confidence interval of the ICC, since each rater would rate 3 trainees on 14 different elements, which gives a total of 462 data points when 11 raters are employed. This is well above the 140 required data points for a narrow confidence interval, when inter-rater reliability of 0.7 or above is expected [27].

### Rater familiarisation with PhaBS

Two researchers (S. E. S. and S. M. S.) conducted a 30-min PhaBS familiarisation session with all raters (in two separate groups). The session was conducted online via Microsoft Teams. It included a brief introduction to the structure of a typical BMS and a specific overview of PhaBS. We encouraged raters to read the situation awareness category of PhaBS and then watch a short situation-comedy-based video clip. Raters scored the sit-com character, using the situation awareness category of PhaBS. We repeated this process, using different video clips, for each of the other categories. The purpose of the activity was to increase familiarity with the PhaBS-positive and -negative behaviours and scoring system and provide an opportunity for questions and discussion.

### Data collection

Immediately following the 30-min familiarisation session, raters were asked to score the three videos of simulated scenarios described above. They were asked to score the trainee pharmacists’ behavioural skills while watching the videos but were also allowed a further 3 min after completion of the video to complete their scoring. Questions were not permitted, and raters were asked not to confer. At the end of the session, all raters sent their completed PhaBS score cards to the lead researcher, alongside their demographic data and signed consent forms.

### Data analysis

All data analysis was conducted using IBM SPSS version 29.0.0.

### Data analysis: Aim 1

Inter-rater reliability of PhaBS was determined using the intra-class correlation coefficient (ICC). Table 3 explains the difference between various types of ICC statistic and explains the reasons that we chose to perform a two-way random, absolute agreement, single-measures ICC.

**Table 3:** Explanation of the types of ICC (adapted from Landers, 2015 [28]), with justification of the type of ICC used in this study

Type of ICC	Explanation of the difference	Type used in this study	Justification
One-way random (ICC 1), two-way random (ICC 2) or two-way mixed (ICC 3)	One-way random assumes that there are no consistent raters for all ratees Two-way random assumes consistent raters for all ratees, and the raters are a sample from a larger population. Two-way mixed assumes consistent raters for all ratees, and the raters are a population, not a sample	Two-way random	All raters rated the same ratees. Our raters were a sample from a larger population
Correlation or absolute agreement	Absolute agreement is used when it is important for scores to be the same (such as in academic exams). Correlation is used if, for example, a mean of ratings will be used, and the absolute value is less important	Absolute agreement	Desire to know how well each rater would assess the ratee
Single measures or average measures	The single-measures ICC determines the accuracy of a single rater when used alone. The average-measures ICC determines the accuracy if multiple raters are used	Single measures	Desire to understand the accuracy of a single rater when used alone

Interpretation of ICC results is somewhat subjective, but we chose to use Cicchetti’s scale: ≤ 0.40 poor agreement, 0.40–0.59 fair agreement, 0.60–0.74 good agreement, and ≥ 0.75 excellent agreement [29].

Other BMS studies which report two-way random, absolute agreement, single-measures ICCs for their scales have reported the following results:

- Medical student non-technical skills (Medi-StuNTS): 0.37 (0.26–0.52) [1]
- Non-technical skills for surgeons (NOTSS): 0.29–0.66 [30]

We therefore pragmatically considered an ICC of 0.4 (fair agreement), to be a reasonable cut-off for inter-rater reliability for PhaBS.

### Data analysis: Aim 2

We assessed rater competence in each of the five areas using the following methods:

### Completeness

The numbers of raters who were able to score every element for every simulation participant were compared using Pearson’s chi-squared test.

### Agreement with an expert rater

An expert rater marked the pharmacist in each simulated scenario using the PhaBS marker system, at the same time as the other experienced faculty. The expert rater was a clinically experienced pharmacist, with a leadership role in pharmacy simulation, who participates regularly in facilitating simulations for trainee pharmacists. He had a high degree of familiarity with both the performance expectations of trainee pharmacists and the PhaBS marker system.

Agreement with the expert rater was calculated using a weighted kappa. Agreement scores were classified into ‘poor’ (kappa < 0.40) or ‘fair or better’ (≥ 0.40). Numbers of raters with ‘poor’ versus ‘fair or better’ were compared using Pearson’s chi-squared test.

### Ability to rank performance

The number of raters in each group able to rank the three performances in the correct order (good performance, mediocre performance, and poor performance) was compared using Pearson’s chi-squared test.

### Stringency

We totalled the scores given by each rater for each simulation participant and used these to calculate a mean score per simulation participant. We assessed raters as hawks (extremely stringent) if their mean scores were two standard deviations or more below the mean and as doves (extremely lenient) if their mean scores were two standard deviations or more above the mean [15].

We used Pearson’s chi-squared test to assess the differences in numbers of extremely stringent or lenient assessors in each group.

### Ability to identify appropriate range of behaviours within performance

We were interested in assessing for the halo effect, in which raters fail to adequately differentiate between different behaviours within a single performance. There is no standard way to calculate this, but one way is to compare the standard deviations across the performances [31].

We hoped that in calculating the spread of scores that each rater gave to each simulation participant, we would be able to assess whether raters could identify positive behaviours within an overall poor performance and negative behaviours within an overall good performance.

We were interested in the spread of scores given by each rater to each simulation performance. For a single rater, we calculated the standard deviation of scores given to performance 1, the standard deviation of the scores given to performance 2, and the standard deviation of the scores given to performance 3. We then summed these three scores to give an overall ‘spread score’.

We repeated this for all raters. We then looked more closely at the scores given by raters who fell outside of one standard deviation from the mean ‘spread score’ for all raters, noting differences between the scores they gave versus the expert rater score, in order to decide whether or not they should be deemed competent.

We assessed the difference between number of raters with low, high, or normal ‘spread scores’ using Pearson’s chi-squared test.

### Aggregate score

We compared the number of raters fulfilling all five of the above criteria in each group, using Pearson’s chi-squared test.

### Correction for multiple comparisons

We made six comparisons between the experienced faculty group and near-peer group. Using a Bonferroni correction for multiple comparisons, we considered the results to be statistically significant if *p* < 0.0083.

## Results

### Demographics

We recruited 22 raters, including pharmacists from all 3 regions of Scotland. Their demographic data are given in Table 4.

**Table 4:** Demographic data of participants. Lengths of time are given as means, with range in brackets

	**Experienced faculty group**	**Near-peer group**
Number of participants	Nine participants	Thirteen participants
Length of clinical experience	18.4 years (8–27 years)	2 years (8 months–3 years)
Length of pharmacy education experience	8.3 years (1.5–17 years)	0 year (no participants with any experience)
Length of pharmacy simulation faculty experience	3.5 years (1–17 years)	0 year (no participants with any experience)

### Aim 1: Inter-rater reliability of PhaBS

For the experienced faculty group, all data were completed. The single-measure ICC for this group was 0.60 (good agreement), with confidence intervals of 0.48 (fair agreement) to 0.72 (good agreement).

**Fig. 1:**
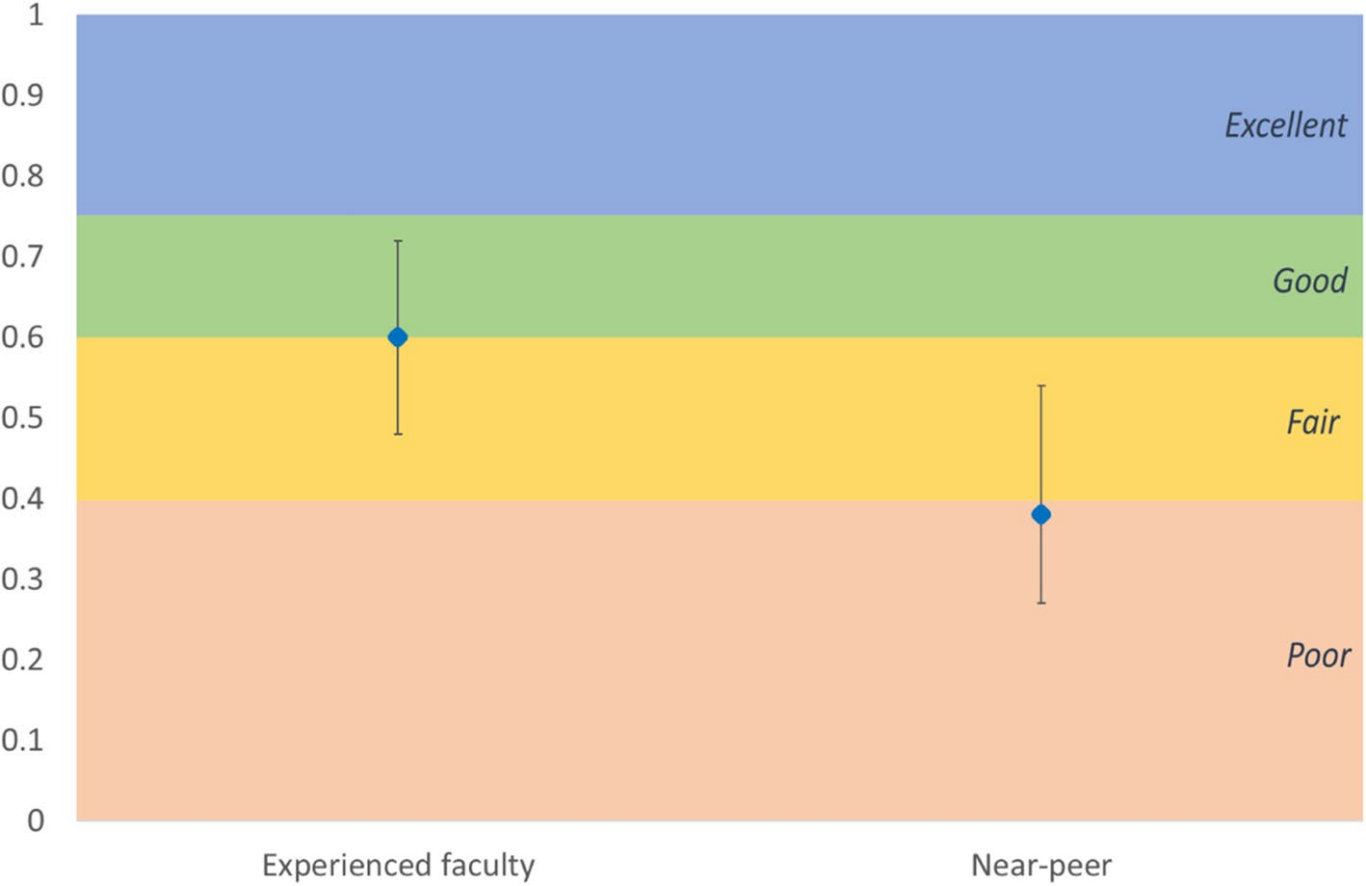
ICC with confidence intervals for the experienced faculty raters versus the near-peer raters

For the near-peer group, there were several rows with incomplete data. Eight out of 42 rows of data were therefore excluded from the analysis (as ICC requires a complete set of data in each row). The single-measures ICC for this group was 0.38 (poor agreement), with confidence intervals of 0.27 (poor agreement) to 0.54 (fair agreement). The differences between in the two groups are shown in the graph in Fig. 1.

### Aim 2: Rater competence in each area

**Fig. 2:**
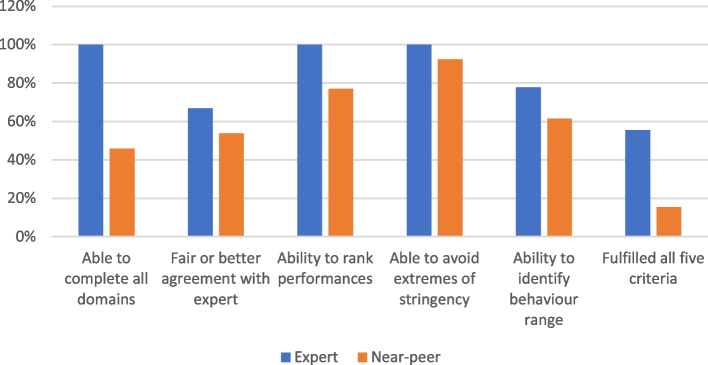
Percentage of experts and near peers who score well for completeness, agreement with experts, ability to rank performances, ability to avoid extremes of stringency, ability to identify an appropriate behaviour range and aggregate score (ability to fulfil all five criteria)

Competence in each of the five attributes, alongside ability to fulfil all five criteria, is shown in Fig. 2.

The only statistically significant difference between the groups was completeness of data, with 9/9 (100%) of experts versus 6/13 (46.2%) able to give scores for every element (*p* = 0.0077).

### Identifying a behaviour range

We interrogated the data to understand which raters would be deemed incompetent excluding those who had a spread score of more than one standard deviation from the mean.

Those with extremes of variability score are discussed in Table 5.

**Table 5:** Analysis of the exclusion attributed to the variability score and whether this was warranted

Rater	Score	Interpretation	Previously excluded?	If not, exclusion based on variability score warranted?
Experienced faculty EF1	1.13	Low variability (halo effect)	No	For the overall poor simulation performance, the rater had awarded only ‘poor’ scores for every element, in contrast with all other raters. Exclusion warranted
Experienced faculty EF8	1.29	Low variability (halo effect)	Yes — poor agreement with the expert rater	
Near-peer NP10	1.41	Low variability (halo effect)	No	This rater gave only ‘poor’ or ‘marginal’ scores to both the overall poor and overall mediocre simulation performances, despite most other raters finding examples of ‘good’ or ‘acceptable’ behaviours. Exclusion warranted
Near-peer NP1	3.80	High variability	No	This rater awarded six inappropriate ‘poor’ scores to the overall good performance, in contrast with all other raters. Exclusion warranted
Near-peer NP6	3.10	High variability	Yes — data incomplete and poor agreement with expert rater	
Near-peer NP8	3.16	High variability	Yes — data incomplete	
Near-peer NP12	3.57	High variability	Yes — data incomplete, poor agreement with expert rater and inability to rank	

Note that while we were originally intending to assess the halo effect (low score spread), further interrogation of the data led us to believe that high variability was a further sign of aberrant examiner behaviour which warranted a closer look at such raters.

### Results summary

In summary, within the experienced faculty group, all raters were able to rank performance and avoid extreme stringency or leniency. Six out of the nine raters were also able to achieve fair or better agreement with the expert rater. A group of seven were able to identify an appropriate behaviour range. Overall, five out of nine raters were competent in all five areas after a single familiarisation session.

Within the near-peer group, a large majority were able to rank the performance and avoid extreme stringency or leniency. When identifying a behaviour range, only 6 out of 13 were able to identify an appropriate range. Seven out of 13 showed fair or better agreement with the expert rater. Overall, 2 out of 13 fulfilled all 5 criteria.

The only statistically significant difference between the two groups was that of data completeness. All of the experienced faculty raters were able to provide a complete data set, versus less than half of the near-peer raters.

## Discussion

This study has investigated the inter-rater reliability of PhaBS when used by experienced faculty raters, versus near-peer raters. The experienced faculty group showed at least fair-good agreement, whereas the near-peer group was poor-fair. Based on this information, we can conclude that PhaBS has inter-rater reliability that is at least as good as other BMS, when used by experienced faculty.

However, this information only tells one part of a story. On interrogating the data more closely, it is clear that there is considerable variation in rater competence. Not all raters can be deemed competent after only a brief familiarisation with the tool. Assessing the competence of the raters based on completeness, agreement with an expert rater, ability to rank performances, and stringency led to an assessment of incompetence for 3/9 experienced faculty raters and 11/13 near-peer raters.

The above measures alone were not sufficient to fully exclude all incompetent raters. In this study, we have produced a new assessment of the ability to identify an appropriate behaviour range within a performance (the spread score). This led to an assessment of incompetence for a further two experienced faculty raters, on the grounds of poor variability (giving similar scores across all elements regardless of the observed behaviour range, otherwise known as the halo effect). It also led to an assessment of incompetence for a near-peer rater, who, despite showing fair agreement with the expert, had awarded a high number of aberrant ‘poor’ scores. We believe that this extra assessment of rater competence is helpful for identifying those raters whose scores should be looked at more closely.

Notably, the experienced faculty were much better at completing all scores than the near-peer assessors. We hypothesise two possible reasons for this. This first concerns the cognitive load placed on the two groups of raters. Cognitive load theory purports that we are only able to hold a small amount of information in our working memory at one time [32]. Experienced faculty are more familiar with using rating systems in general and may have been more familiar with some of the terms in the marker system; thus, their cognitive load may have been lower. However, against this hypothesis is the fact that not all experienced faculty were able to identify an appropriate behaviour range, suggesting that they could not give the whole marker system their full attention. An explanation that is perhaps more likely is that experienced faculty were using system 1 thinking (also known as type 1 reasoning) to help them complete the ratings. System 1 thinking is a rapid process that uses intuition, whereas system 2 thinking is a slower, more effortful analytic process [33]. It has previously been suggested that raters use of system 1 versus system 2 thinking might affect their judgements [34]. The hypothesis that the experienced faculty raters may be using more system 1 thinking than the near-peer assessors is supported by the fact that, unlike the near-peer raters, all experienced faculty raters were able to complete all ratings and rank the performances in order. In fact, one member of experienced faculty gave one performance only ‘poor’ ratings throughout, suggesting that the rater attended to their intuition rather than faithfully utilising the marker system.

Raters may have reverted to intuitive thinking in response to the high mental workload required to rate performances using a marker system. Mental workload has previously been shown to be high for objective structured clinical examinations (OSCEs) [35]. Given that OSCEs are checklist based, BMS (which require raters to rate each individual item) may require an even higher mental workload, though this has not been studied. Possible mechanisms to reduce mental workload include rater training [36, 37] or task simplification [36–38]. For example, if using PhaBS for summative assessment, several raters could each mark a single performance, and each rater could intentionally focus on a different category within the marker system. This would reduce the number of items for each rater to mark, which may be helpful given that longer checklists are associated with a higher chance of reverting to global assessments [39]. The impact of facilitator training, or task simplification, on PhaBS rater competence could usefully form the basis of a further study.

Ten years ago, Dietz et al. called for established standards for the testing and reporting of psychometric evidence of BMS [40], but despite their increased use across a variety of disciplines, there is no consensus on reliability testing of these tools. Throughout the BMS literature, there are examples of inter-rater reliability, often measured using ICC [1, 12, 30, 41]. The ICC scores are often very low when the single-measure ICCs are reported, suggesting that it is difficult to achieve good inter-rater reliability for BMS. This study has shown that even when the ICC is in the ‘good’ range, it does not follow that every rater is competent. We would suggest that for BMS, instead of seeking high levels of inter-rater reliability, we should instead focus on methods for assessing rater competence. When doing so, we should include within our assessment the ability to rank actual observed behaviours, rather than giving overall intuitive scores. Rater competence assessments may also be a helpful way to better understand responses to rater training.

### Strengths and limitations

This inter-rater reliability study introduced raters to a new BMS, PhaBS, using a brief familiarisation session that could easily be replicated on a larger scale. This study therefore gleaned data from naïve users of the tool, which may more closely mirror real-life introductions to such a tool, rather than a lengthy training session.

Our study’s main aim was to assess the inter-rater reliability, and a secondary aim was to assess rater competence. We based our sample on the number needed for inter-rater reliability assessments. As such, our study was insufficiently powered to detect differences in some of the competence attributes between the two groups. Additionally, Pearson’s chi-squared test may not be as reliable with small sample sizes.

Our scenarios were scripted, which is common in BMS inter-rater reliability studies [2, 30, 42]. This allowed us to test raters’ competence in assessing a range of performances. However, it would be interesting to study the ICCs with unscripted scenarios, to more closely reflect real-world use.

A potential confounding factor was that the experienced faculty and near-peer groups undertook their familiarisation sessions separately. We attempted to ensure that the training was as similar as possible, with the same facilitator, same script, and same amount of time allowed. As researchers, we had no vested interest in finding either the experienced faculty or near-peer group to be better, and so did not risk any accidental bias in delivering the session. Nevertheless, in an ideal study, all raters would have attended the same session together.

### Further work

In this study, we created a ‘spread score’, which was based on previous methods for assessing the halo effect [31]. This method was somewhat validated within our small data set, but it would be useful to analyse larger numbers of raters to validate this method. A larger data set would also help us to identify differences between competence scores for experienced faculty and near-peer assessors, although arguably, given that less than half the near-peer assessors were able to complete the assessment, and we could not recommend that they use it without additional training.

It would be very helpful to study whether rater training could improve rater competence, noting that previous work has been unable to show any improvement in competence as a result of rater training for assessments such as the mini-CEX [43] or ANTS [12]. It would also be interesting to conduct a qualitative study, to explore how raters use BMS. Similar studies have been illuminating for studying OSCE examiners [44, 45], but have not been replicated in BMS raters.

Regarding the PhaBS tool, this study has provided evidence for inter-rater reliability (a subset of internal structure validity evidence). Content validity evidence for this tool has been presented elsewhere. Future studies could aim to provide further validity evidence for this tool according to Messick’s framework, including additionally internal structure evidence, as well as response process, relationship with other variables, and consequences evidence [6, 7].

We would suggest that within simulation, PhaBS may be helpful for scenario design, to provide a common language within debriefing conversations, and as an observer tool. Further work could usefully focus on whether PhaBS provides benefits within any of these domains. PhaBS may also be helpful as a research tool, though if used as such each rater’s competence should first be assessed using the competence framework suggested within this paper.

## Conclusions

Experienced faculty have acceptable inter-rater reliability when using PhaBS. However, acceptable inter-rater reliability does not mean that all raters are competent. When considering using BMS for summative assessment or research, we need to change the conversation and look beyond inter-rater reliability to start asking important questions about rater competence. This will help us to provide fair and accurate assessments for learners, as well as provide us with useful information about rater training programmes, and provide individual raters with meaningful feedback.

## Supplementary Information

Additional file 1: Appendix: Pharmacy simulation scenarios description.

## Data Availability

Raw data is available on request from the authors.

## Abbreviations

ANTS: Anaesthetists’ non-technical skills
BMS: Behavioural marker system
ICC: Intra-class correlation co-efficient
Medi-StuNTS: Medical students non-technical skills
OSCE: Objective structured clinical examination
NOTSS: Non-technical skills for surgeons
PhaBS: Pharmacists’ behavioural skills
UK: United Kingdom

## References

[1] Clarke B, Smith SE, Phillips EC, Hamilton A, Kerins J, Tallentire VR. Reliability of assessment of medical students’ non-technical skills using a behavioural marker system: does clinical experience matter? BMJ Simul Technol Enhanc Learn. 2021;7:285. 35515716 10.1136/bmjstel-2020-000705 PMC8936703

[2] Fletcher G, Flin R, McGeorge P, Glavin R, Maran N, Patey R. Anaesthetists’ non-technical skills (ANTS): evaluation of a behavioural marker system. Br J Anaesth. 2003;90:580–8. 12697584 10.1093/bja/aeg112

[3] Yule S, Flin R, Paterson-Brown S, Maran N, Rowley D. Development of a rating system for surgeons’ non-technical skills. Med Educ. 2006;40:1098–104. 17054619 10.1111/j.1365-2929.2006.02610.x

[4] Hamilton AL, Kerins J, MacCrossan MA, Tallentire VR. Medical students’ non-technical skills (Medi-StuNTS): preliminary work developing a behavioural marker system for the non-technical skills of medical students in acute care. BMJ Simul Technol Enhanc Learn. 2019;5. 10.1136/bmjstel-2018-000310 PMC8936547 35514943

[5] American Psychological Association. APA Dictionary of Psychology. American Psychological Association. 2007. Available from: https://dictionary.apa.org/interrater-reliability. Cited 2024 Dec 4

[6] Validity MS. In: Linn RL, editor. Educational measurement. 3rd ed. New York: American Council on Education and Macmillan; 1989. p. 13–103.

[7] Cook DA, Zendejas B, Hamstra SJ, Hatala R, Brydges R. What counts as validity evidence? Examples and prevalence in a systematic review of simulation-based assessment. Adv Health Sci Educ. 2014;19:233–50. 10.1007/s10459-013-9458-4. 10.1007/s10459-013-9458-4 23636643

[8] Harlen W, James M. Assessment and learning: differences and relationships between formative and summative assessment. Assess Educ. 1997;4:365–79. 10.1080/0969594970040304.

[9] Downing SM. Reliability: on the reproducibility of assessment data. Med Educ. 2004;38:1006–12. 15327684 10.1111/j.1365-2929.2004.01932.x

[10] Schleicher DJ, Day D V. A cognitive evaluation of frame-of-reference rater training: content and process issues. Organ Behav Hum Decis Process 1998;73:76–101. Available from: https://www.sciencedirect.com/science/article/pii/S0749597898927510 10.1006/obhd.1998.2751 9705795

[11] Yeates P, O’Neill P, Mann K, Eva K. Seeing the same thing differently. Adv Health Sci Educ. 2013;18:325–41. 10.1007/s10459-012-9372-1. 10.1007/s10459-012-9372-1 22581567

[12] Graham J, Hocking G, Giles E. Anaesthesia non-technical skills: can anaesthetists be trained to reliably use this behavioural marker system in 1 day? Brit J Anaesthesia. 2010;104:440–5. 10.1093/bja/aeq032. 10.1093/bja/aeq032 20190257

[13] Allison R, Katona C. Audit of oral examinations in psychiatry. Med Teach. 1992;14:383–9. 10.3109/01421599209018860. 1293467 10.3109/01421599209018860

[14] Newble DI, Hoare J, Sheldrake PF. The selection and training of examiners for clinical examinations. Med Educ. 1980;14:345–9. 10.1111/j.1365-2923.1980.tb02379.x. 7432220 10.1111/j.1365-2923.1980.tb02379.x

[15] Bartman I, Smee S, Roy M. A method for identifying extreme OSCE examiners. Clin Teach. 2013;10:27–31. 23294740 10.1111/j.1743-498X.2012.00607.x

[16] Thorndike EL. A constant error in psychological ratings. J Appl Psychol. 1920;4:25–9.

[17] Smith SE, Kerins J, McColgan-Smith S, Stewart F, Power A, Mardon J, et al. The development of a marker system for pharmacists’ behavioural skills. Int J Pharm Pract. 2023;31:520–7. 37452687 10.1093/ijpp/riad041

[18] Govaerts MJB, Van de Wiel MWJ, Schuwirth LWT, Van der Vleuten CPM, Muijtjens AMM. Workplace-based assessment: raters’ performance theories and constructs. Advances in Health Sciences Education. 2013;18:375–96. 10.1007/s10459-012-9376-x. 22592323 10.1007/s10459-012-9376-x PMC3728456

[19] Govaerts MJB, Schuwirth LWT, Van der Vleuten CPM, Muijtjens AMM. Workplace-based assessment: effects of rater expertise. Adv Health Sci Educ. 2011;16:151–65. 10.1007/s10459-010-9250-7. 10.1007/s10459-010-9250-7 PMC3068251 20882335

[20] Kerins J, Smith SE, Phillips EC, Clarke B, Hamilton AL, Tallentire VR. Exploring transformative learning when developing medical students’ non-technical skills. Med Educ. 2020;54:264–74. 31954079 10.1111/medu.14062

[21] Watts PI, McDermott DS, Alinier G, Charnetski M, Ludlow J, Horsley E, et al. Healthcare simulation standards of best practiceTM simulation design. Clin Simul Nurs. 2021;58:14–21.Available from: https://www.sciencedirect.com/science/article/pii/S1876139921000967

[22] Boet S, Bould MD, Bruppacher HR, Desjardins F, Chandra DB, Naik VN. Looking in the mirror: self-debriefing versus instructor debriefing for simulated crises*. Crit Care Med. 2011;39. Available from: https://journals.lww.com/ccmjournal/fulltext/2011/06000/looking_in_the_mirror__self_debriefing_versus.21.aspx 10.1097/CCM.0b013e31820eb8be 21317645

[23] Yule S, Flin R, Maran N, Youngson G, Mitchell A, Rowley D, et al. Debriefing surgeons on non-technical skills (NOTSS). Cognition, Technology & Work. 2008;10:265–74. 10.1007/s10111-007-0085-9.

[24] O’Regan S, Molloy E, Watterson L, Nestel D. Observer roles that optimise learning in healthcare simulation education: a systematic review. Adv Simul. 2016;1:4. 10.1186/s41077-015-0004-8. 10.1186/s41077-015-0004-8 PMC5796608 29449973

[25] Chief Pharmaceutical Officers and UK Pharmacy Regulators. Reforms to initial education and training of pharmacists. https://www.pharmacyregulation.org/sites/default/files/document/joint_letter_from_cphos_and_uk_pharmacy_regulators_28_july_2020.pdf. 2020.

[26] Cheng A, Auerbach M, Hunt EA, Chang TP, Pusic M, Nadkarni V, et al. Designing and conducting simulation-based research. Pediatrics. 2014;133:1091–101. 24819576 10.1542/peds.2013-3267

[27] Saito Y, Sozu T, Hamada C, Yoshimura I. Effective number of subjects and number of raters for inter-rater reliability studies. Stat Med. 2006;25:1547–60. 16143966 10.1002/sim.2294

[28] Landers RN. Computing intraclass correlations (ICC) as estimates of interrater reliability in SPSS. The Winnower. 2015;2.

[29] Cicchetti DV. Guidelines, criteria, and rules of thumb for evaluating normed and standardized assessment instruments in psychology. Psychol Assess. 1994;6:284.

[30] Yule S, Flin R, Maran N, Rowley D, Youngson G, Paterson-Brown S. Surgeons’ non-technical skills in the operating room: reliability testing of the NOTSS behavior rating system. World J Surg. 2008;32:548–56. 18259809 10.1007/s00268-007-9320-z

[31] Cooper WH. Ubiquitous halo. Psychol Bull. 1981;90:218.

[32] Sweller J. Cognitive load during problem solving: effects on learning. Cogn Sci. 1988;12:257–85.

[33] Kahneman D. Thinking, fast and slow. New York: Farrar, Strauss and Giroux; 2011.

[34] Wood TJ. Mental workload as a tool for understanding dual processes in rater-based assessments. Adv Health Sci Educ. 2013;18:523–5. 10.1007/s10459-012-9396-6. 10.1007/s10459-012-9396-6 22886141

[35] Byrne A, Tweed N, Halligan C. A pilot study of the mental workload of objective structured clinical examination examiners. Med Educ. 2014;48:262–7. 24528461 10.1111/medu.12387

[36] Wilby KJ, Paravattil B. Cognitive load theory: implications for assessment in pharmacy education. Res Soc Adm Pharm. 2021;17:1645–9. Available from: https://www.sciencedirect.com/science/article/pii/S1551741120312341 10.1016/j.sapharm.2020.12.009 33358136

[37] Paravattil B, Wilby KJ. Optimizing assessors’ mental workload in rater-based assessment: a critical narrative review. Perspect Med Educ. 2019;8:339–45. 31728841 10.1007/s40037-019-00535-6 PMC6904389

[38] Tavares W, Eva KW. Exploring the impact of mental workload on rater-based assessments. Adv Health Sci Educ. 2013;18:291–303. 10.1007/s10459-012-9370-3. 10.1007/s10459-012-9370-3 22484964

[39] Kogan JR, Conforti LN, Bernabeo E, Iobst W, Holmboe E. How faculty members experience workplace-based assessment rater training: a qualitative study. Med Educ. 2015;49:692–708. 10.1111/medu.12733. 26077217 10.1111/medu.12733

[40] Dietz AS, Pronovost PJ, Benson KN, Mendez-Tellez PA, Dwyer C, Wyskiel R, et al. A systematic review of behavioural marker systems in healthcare: what do we know about their attributes, validity and application? BMJ Qual Saf. 2014;23:1031. Available from: http://qualitysafety.bmj.com/content/23/12/1031.abstract 25157188 10.1136/bmjqs-2013-002457

[41] Nunnink L, Foot C, Venkatesh B, Corke C, Saxena M, Lucey M, et al. High-stakes assessment of the non-technical skills of critical care trainees using simulation: feasibility, acceptability and reliability. Crit Care Resusc. 2014;16:6–12. 24588430

[42] Spanager L, Beier-Holgersen R, Dieckmann P, Konge L, Rosenberg J, Oestergaard D. Reliable assessment of general surgeons’ non-technical skills based on video-recordings of patient simulated scenarios. The American Journal of Surgery. 2013;206:810–7. 23871323 10.1016/j.amjsurg.2013.04.002

[43] Cook DA, Dupras DM, Beckman TJ, Thomas KG, Pankratz VS. Effect of rater training on reliability and accuracy of mini-CEX scores: a randomized, controlled trial. J Gen Intern Med. 2009;24:74–9. 10.1007/s11606-008-0842-3. 19002533 10.1007/s11606-008-0842-3 PMC2607488

[44] Scully C, Kelly M, Lysaght Z, O’Leary M. The cognitive processes employed by undergraduate nursing OSCE assessors: a qualitative research study. Nurse Educ Today. 2024;134:106083. 38183907 10.1016/j.nedt.2023.106083

[45] Hyde S, Fessey C, Boursicot K, MacKenzie R, McGrath D. OSCE rater cognition – an international multi-centre qualitative study. BMC Med Educ. 2022;22:6. 10.1186/s12909-021-03077-w. 34980099 10.1186/s12909-021-03077-w PMC8721185

