# Supplementary material for: Beyond reliability: assessing rater competence when using a behavioural marker system
Source: Adv Simul (Lond). 2024 Dec 31;9:55. doi: 10.1186/s41077-024-00329-9 (PMC11687013; doi:10.1186/s41077-024-00329-9)
Supplement: Supplementary file 1 — Additional file 1: Appendix: Pharmacy simulation scenarios description. [file 41077_2024_329_MOESM1_ESM.docx]

**Appendix: Pharmacy simulation scenarios description**

Cheng et al.’s reporting guidelines are followed below, in describing the simulation course. This template table is available here: Cheng A, Kessler D, Mackinnon R, Chang TP, Nadkarni VM, Hunt EA, Duval-Arnould J, Lin Y, Cook DA, Pusic M, Hui J. Reporting guidelines for health care simulation research: extensions to the CONSORT and STROBE statements. Simulation in Healthcare. 2016 Aug 1;11(4):238-48.

| **Elements** | **Subelements** | **Pharmacy simulation scenarios** |
| --- | --- | --- |
| Participant orientation | Orientation to the simulator | The participant actors were familiar with the simulator due to previous interactions as both learners and faculty. |
|  | Orientation to the environment | As above. |
| Simulator type | Simulator make and model | Laerdal SimMan |
| Simulator type | Simulator functionality | Interactive components of the simulator include a voice provided by a microphone in the control room connected to a speaker in the head of SimMan. Pupils are reactive to light and can be changed in the control room. Pulses can be palpated. Heart and breath sounds can be auscultated and altered within the control room. |
| Simulation environment | Location | Simulation centre (University Hospital Ayr) |
|  | Equipment | The available equipment included all emergency equipment normally available on a ward: Oxygen masks, airway adjuncts, peripheral venous cannulae, intravenous fluids and giving sets, a defibrillator, simulated emergency drugs. |
|  | External stimuli | None |
| Simulation event / scenario | Event description | Scenarios were designed around specific learning objectives, with trigger points to move to the next stage of the scenario. |
|  | Learning objectives | See below for learning objectives for each scenario. |
|  | Group vs. individual practice | Scenarios focused on the performance of a single trainee pharmacist. |
| Simulation event / scenario | Use of adjuncts | Not required. |
|  | Facilitator / operator characteristics | The facilitator was a member of the research team, with formal training in simulation course design and delivery, and a leadership role in pharmacy simulation in Scotland. |
|  | Pilot testing | These scenarios had been previously delivered to trainee pharmacists across Scotland as part of their standard training. |
|  | Actors / confederates / standardized / simulated patients | Embedded professionals included:   1. A simulation technician acting in the role of an advanced nurse practitioner. 2. A trainee pharmacist acting in the role of a patient. |
| Instructional design (for educational interventions) or exposure (for simulation as investigative methodology) | Duration | Each scenario lasted between five and six minutes. |
|  | Timing | Scenarios were filmed approximately two months prior to data collection. |
|  | Frequency / repetitions | Scenarios were not repeated. |
|  | Clinical variation | Three different acute care scenarios were used:   1. An elderly patient with urosepsis and Parkinson’s disease 2. A patient with severe acute asthma 3. A patient with epilepsy who had suffered a stroke |
|  | Standards / assessment | Raters assessed the video-recorded scenarios using PhaBS. |
|  | Adaptability of intervention | No adaptations. |
|  | Range of difficulty | Scenarios were pitched at a range designed to challenge a trainee pharmacist, and expose positive and negative behavioural skills. |
|  | Nonsimulation interventions and adjuncts | None |
|  | Integration | None (investigative methodology, not an educational intervention) |
| Feedback and / or debriefing | Source | None – see above. |
|  | Duration | Not applicable |
|  | Facilitator presence | Not applicable |
|  | Facilitator characteristics | Not applicable |
|  | Content | Not applicable |
|  | Structure / method | Not applicable |
|  | Timing | Not applicable |
|  | Video | Not applicable. |
|  | Scripting | Not applicable |

A further description of each scenario is given below. The scenarios are usually undertaken during an inter-professional simulation, with a fifth year medical student and trainee pharmacist. In this study, an embedded professional playing the role of an advanced nurse practitioner took the place of a competent fifth year medical student. A recently qualified pharmacist played the role of a trainee pharmacist, but her responses were scripted in order to deliberately display examples of positive and negative behavioural skills.

**Scenario 1: Urosepsis in Parkinson’s disease**

***Synopsis:*** A 74-year-old man is brought into the emergency department with non-specific complaints. He is found to have urosepsis. His usual medicines include Parkinson’s medications, which he is unable to take by mouth.

***Learning Objectives***

- Demonstrate team working skills and understand the different roles within the team
- Demonstrate appropriate communication skills with members of the multidisciplinary team
- Demonstrate a structured approach to managing sepsis
- Demonstrate a structured approach to managing Parkinson’s when patient is nil by mouth

In this scenario, the trainee pharmacist displays a variety of ‘good’ and ‘excellent’ behavioural skills. An example was challenging the advanced nurse practitioner’s antibiotic prescribing choice, and directing her to a more appropriate one.

**Scenario 2: Acute severe asthma**

***Synopsis:*** A 20-year-old man is brought in by ambulance. He is wheezy and short of breath. He is found to have severe acute asthma, and requires a magnesium sulfate infusion.

***Learning Objectives***

- Demonstrate team working skills and understand the different roles within the team
- Demonstrate appropriate communication skills with members of the multidisciplinary team
- Demonstrate a structured approach to commencing treatment for asthma exacerbation

In this scenario, the trainee pharmacist displays some ‘good’ and ‘acceptable’ behaviours, but some ‘marginal’ or ‘poor’ behaviours. An example of a ‘good’ behaviour was suggesting a magnesium sulfate infusion as the next step in treatment. An example of a ‘poor’ behaviour was attempting to take a lengthy history despite the patient being clearly unwell.

**Scenario 3: Acute stroke in a patient with epilepsy**

***Synopsis*** A 74-year-old man is brought in by ambulance with a ‘funny turn’. He is found to have an acute stroke, and is unable to swallow his usual epilepsy medications.

***Learning Objectives***

- Demonstrate team working skills and understand the different roles within the team
- Demonstrate appropriate communication skills with members of the multidisciplinary team
- Demonstrate an understanding of the immediate management of acute stroke
- Demonstrate an understanding of medication rationalisation in patient who is nil by mouth

In this example, the trainee pharmacist displays mainly ‘poor’ or ‘marginal’ behaviours, with some ‘acceptable’ behaviours. An example of a ‘poor’ behaviour was failure to prioritise finding an appropriate route by which to deliver the anticonvulsants.
